# Fluid dynamic induced break-up during volcanic eruptions

**DOI:** 10.1038/s41467-019-11750-4

**Published:** 2019-08-23

**Authors:** T. J. Jones, C. D. Reynolds, S. C. Boothroyd

**Affiliations:** 1 Department of Earth, Environmental and Planetary Sciences, Rice University, 6100 Main Street, Houston, TX 77005 USA; 2 Department of Earth Sciences, Durham University, Lower Mountjoy, South Road, Durham, DH1 3LE UK; 3 School of Chemistry, The University of Birmingham, Edgbaston, Birmingham, B15 2TT UK; 4 Department of Chemistry, Durham University, Lower Mountjoy, South Road, Durham, DH1 3LE UK

**Keywords:** Natural hazards, Volcanology

## Abstract

Determining whether magma fragments during eruption remains a seminal challenge in volcanology. There is a robust paradigm for fragmentation of high viscosity, silicic magmas, however little is known about the fragmentation behaviour of lower viscosity systems—the most abundant form of volcanism on Earth and on other planetary bodies and satellites. Here we provide a quantitative model, based on experiments, for the non-brittle, fluid dynamic induced fragmentation of low viscosity melts. We define the conditions under which extensional thinning or liquid break-up can be expected. We show that break-up, both in our experiments and natural eruptions, occurs by both viscous and capillary instabilities operating on contrasting timescales. These timescales are used to produce a universal break-up criterion valid for low viscosity melts such as basalt, kimberlite and carbonatite. Lastly, we relate these break-up instabilities to changes in eruptive behaviour, the associated natural hazard and ultimately the deposits formed.

## Abstract (web summary)

Determining if a volcanic eruption will behave effusively or explosively is crucial for predicting the potential hazard type and for planning effective mitigation. Here, the authors present a universal, fluid dynamic induced, break-up criterion for low viscosity melts.

## Introduction

Establishing when a volcanic eruption will behave effusively, explosively or a combination thereof is of utmost importance to civil protection and hazard mitigation^1–3^. Explosive behaviour produces pyroclastic material building up spatter ramparts, scoria cones and/or tephra blankets, which can potentially lead to air-space closure^4^, whereas effusive behaviour occurs in the form of lava flows and toxic gas emissions^5,6^. Thus, the eruption style directly controls the hazard type, spatial footprint and magnitude. A robust understanding exists for the fragmentation of high viscosity, silicic systems^1,2,7–11^; however, the same theories cannot be applied to the fragmentation of lower viscosity liquids. This knowledge gap is further compounded by the fact that most of the volcanism on Earth^12^ and on other planetary bodies and satellites^13,14^ features low-viscosity liquids. The liquids in question could be of wide-ranging chemical composition from silicate and carbonate melts on Earth to water and aqueous solutions on other planetary bodies.

For evolved silicic systems, where viscosities are high, fragmentation occurs by crossing the rheological glass transition^1^. For a melt in the relaxed state, at any given viscosity high strain rates favour brittle behaviour, whereas lower strain rates favour liquid-like behaviour. For less silicic melts such as basalt, kimberlite and carbonatite (with viscosities of ⪅10^3^ Pa ·s), fragmentation via crossing the glass transition requires very high strain rates >10^4^ s^−1^. Such strain rates are not observed during terrestrial eruption of low-viscosity melts and therefore theories developed for the fragmentation of silicic systems remain unrealistic^15,16^. Fragmentation of low-viscosity melts must occur by a fundamentally different process—fluid dynamic induced break-up—where magma is stretched and pulled apart to form pyroclasts^2,16–19^. Fragmentation by fluid dynamic-induced break-up therefore has the potential to govern eruptive behaviour.

**Fig. 1:**
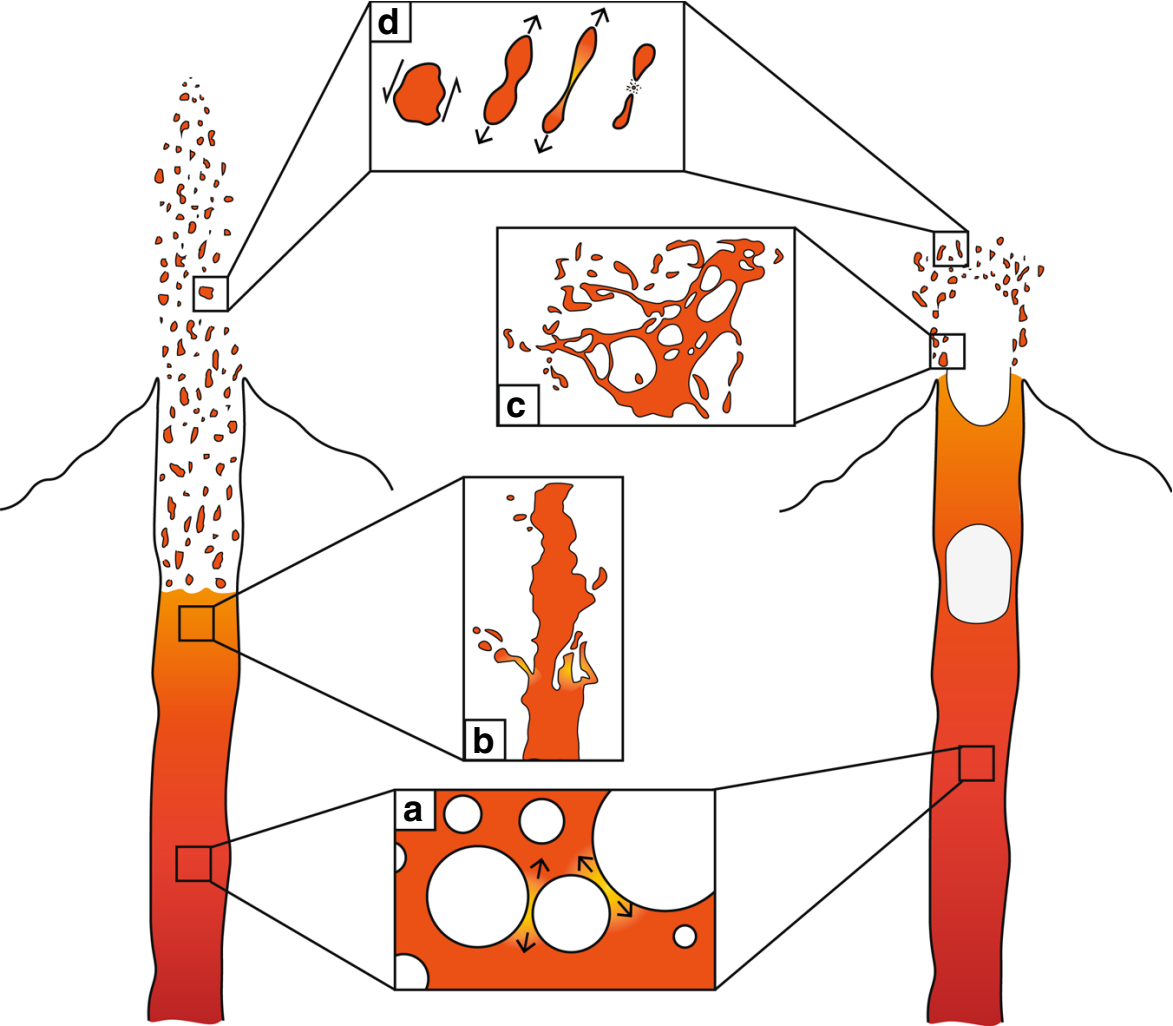
A schematic showing the potential locations where the fluid dynamic break-up of a low-viscosity melt is important. These illustrations are not exhaustive but include **a** filament formation and extension by bubble growth and expansion, **b** instabilities on the edge of a jet, **c** the bursting of bubbles and **d** the continued stretching of pyroclasts in a fountain

During eruption there are many processes where liquid extension plays a central role. Some of these are depicted in Fig. 1. During magma ascent within the conduit, decompression and subsequent bubble growth subject the interstitial melt to extension. If the inertia effect overwhelms the viscous effects, the extending bubble walls continually thin and potentially become subject to break-up^16,20^. Upon exit from the vent, extensional break-up can be caused by fluid dynamic instabilities at the edge of the magma jet^17,19,21–23^. In less energetic cases, the bursting of large gas bubbles or slugs at the free surface leads to magma break-up. This is commonly observed at lava lakes and during Strombolian-style eruptions^24–28^. Lastly, after hot fluidal pyroclasts have been formed they can further fragment during continued extension within the fountain^2,29–33^. We note, however, that this list is not exhaustive; for example, extensional break-up has been observed during the landing of spatter clasts^34,35^ and melt stripping from crystal surfaces^36^. In this study, we focus on the fragmentation behaviour of low-viscosity liquids/melts devoid of bubbles and crystals. The addition of these extra components, through volatile exsolution and/or crystallization, will form a multiphase magma and introduce non-Newtonian effects (as discussed later).

Here, we perform a series of analogue experiments to investigate the extension conditions under which these low-viscosity liquids break-up and propose a universal fragmentation criterion that can be applied to eruptions of low-viscosity liquids worldwide. Under typical eruptive conditions, melts such as basalt, kimberlite and carbonatite break-up via fluid dynamic processes in the form of viscous and capillary instabilities. The timescale that drives break-up is shorter for liquids with a higher surface tension and lower viscosity, making these most prone to break-up. We conclude by defining the conditions under which extensional flow or liquid break-up can be expected and relate this to changes in eruptive behaviour, the associated natural hazard and ultimately the type of deposits generated.

## Results

### Experimental observations

**Fig. 2:**
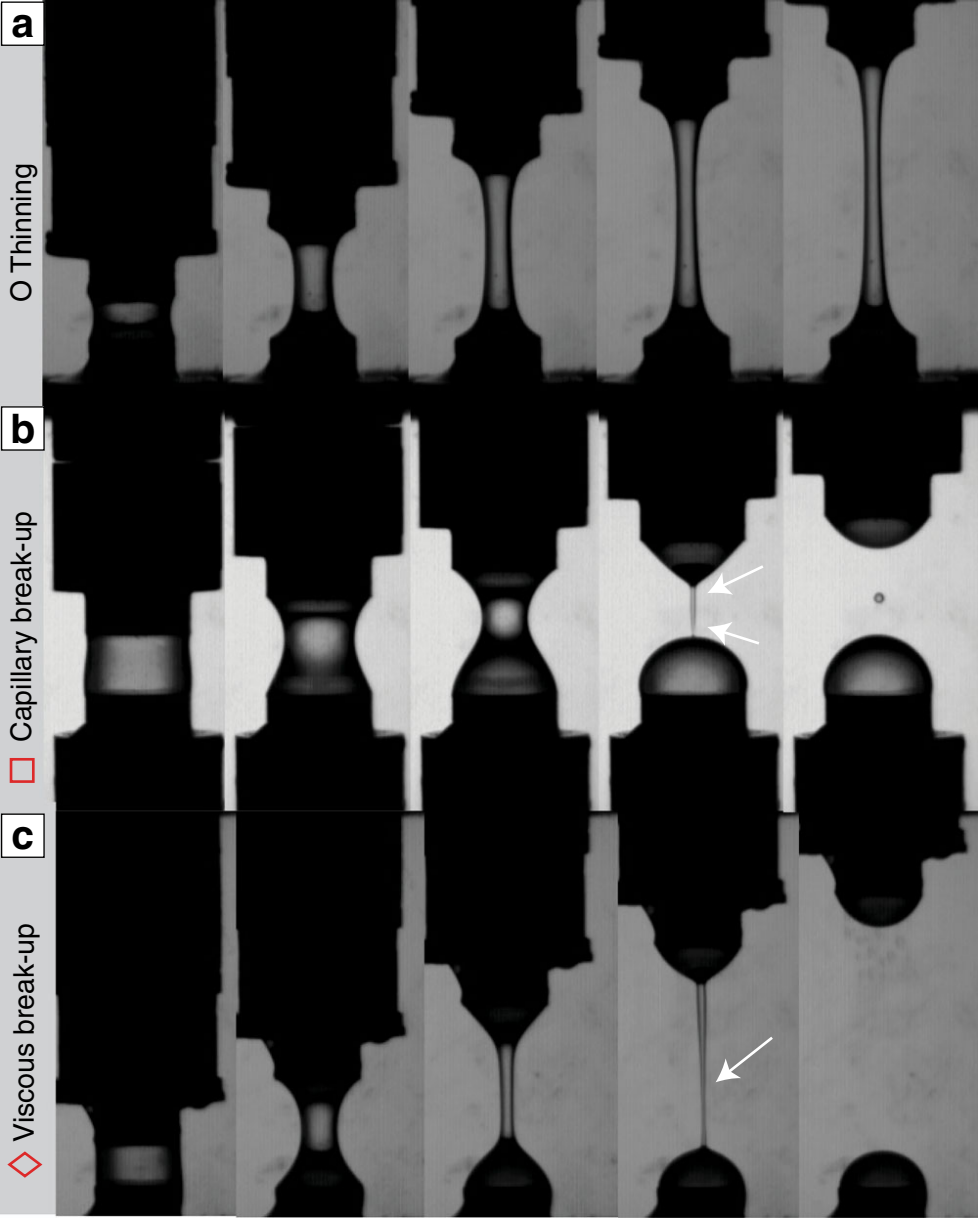
Representative images showing the three styles of behaviour observed. Specifically, these image stills are from experiments that used **a** a 5  wt.% dilution of golden syrup, **b** a 30 wt.% dilution of golden syrup and **c** a 20 wt.% dilution of golden syrup. Images taken at 0, 0.02, 0.04, 0.06 and 0.08 s into the deformation. In experiments a and c the initial droplet had a 4 mm diameter and 2 mm thickness and the sample was extended 14.65 mm over 0.1 s. For experiment b the initial droplet had a 5 mm diameter and 2.5 mm thickness and was extended 6.48 mm over 0.1 s. The white arrows point to the loci of necking

Our experiments subject a liquid held between two parallel plates to vertical extension. Fluid properties including viscosity (92–0.03 Pa·s), surface tension (0.08–0.07 N m^−1^), density (1439–1254 kg m^−3^) and relaxation time were varied using different dilutions of sugar syrup (see Methods). Other experimental variables include the initial liquid dimensions, the extension rate and the total strain. All these variables were chosen such that our experiments cover the same range of dimensionless space and dynamical regimes as expected for natural volcanic eruptions. Every experiment was recorded by high-resolution videography (26 pixels/mm) to document whether the liquid stayed connected as a flowing thinning filament (Fig. 2a) or broke (Fig. 2b, c) during extensional deformation. These two modes of behaviour are referred to as “break-up” and “thinning” hereafter. Furthermore, break-up is observed to occur by two contrasting modes. First, capillary break-up, which is characterized by necking at two points, leaving a central section of the filament, which retracts into a droplet (Fig. 2b). Second, viscous break-up, where the filament thins and necks at a single point between the plates (Fig. 2c). Videos of these experiments can be found in the online supporting information (Supplementary Movies 1, 2 and 3). These modes of break-up were identified using the accompanying videography where possible and later confirmed by the calculation of the dominant timescale (see discussion later; Eq. 3). We found that low fluid viscosities, fast extension rates and thin filament diameters promoted break-up; however, these factors are all interrelated. A full list of experimental conditions and fluid properties can be found in the Supplementary Data 1.

### Characteristic timescales

Following the work of Villermaux^32^, we now introduce the timescales appropriate to the continued elongation of a filament. During the extension of a low-viscosity fluid, the initial volume is stretched into a filament of some thickness, *d*, which reduces with increased extension. After Mckinley and Sridhar^37^, the extension rate, $\dot \gamma$, can be calculated as: $\dot \gamma = - \frac{2}{d}\frac{{\Delta d}}{{\Delta t}},$ where *d* is the filament diameter and Δ*d* is the change in filament diameter over the short time interval, Δ*t* (see Methods for details). The thread or filament is subject to two instability timescales, both acting to break the thread^32,38^. When liquid surface tension and inertia are dominant, break-up is completed within a characteristic capillary instability timescale given by:

1 $$\lambda _{{\mathrm{cap}}} = \sqrt {\frac{{\rho d^3}}{\sigma }},$$

where *ρ* is the fluid density, *σ* is the surface tension and *d* is the filament diameter. However, when viscous forces dominate over inertia, they slow down the break-up process (without suppressing it) such that break-up now occurs on the viscous instability timescale:

2 $$\lambda _{{\mathrm{vis}}} = \frac{{\eta d}}{\sigma },$$

where *η* is the fluid shear viscosity. To determine which of these timescales controls break-up, it is useful to introduce the Ohnesorge number (Oh)—a dimensionless number that has been shown to govern the style of fluid dynamic break-up^22,32,38–40^:

3 $${\mathrm{Oh}} = \frac{{\lambda _{{\mathrm{vis}}}}}{{\lambda _{{\mathrm{cap}}}}} = \frac{\eta }{{\sqrt {\sigma \rho d} }}.$$

Hence, where Oh is >1, the viscous instability timescale dominates, and where Oh is <1, the capillary instability timescale dominates.

## Discussion

Given that a wide variety of interrelated variables control break-up (e.g. low fluid viscosities, fast extension rates and small thread diameters), we introduce a modified dimensionless Deborah number $({\mathrm{De}}^{*})$ in order to provide a universal break-up criterion valid for the extension of a thinning filament. The modified Deborah number is a ratio of the dominant fluid’s instability timescale, *λ*_inst_ to the imposed deformation timescale and *λ*_d_ expressed as:

4 $${\mathrm{De}}^ \ast = \frac{{\lambda _{{\mathrm{inst}}}}}{{\lambda _{\mathrm{d}}}},$$

where *λ*_inst_ = *λ*_vis_ for Oh >1 and *λ*_inst_ = *λ*_cap_ for Oh <1, and $\lambda _{\mathrm{d}} = 1/\dot \gamma$ is the reciprocal of the extension rate, $\dot \gamma$.

**Fig. 3:**
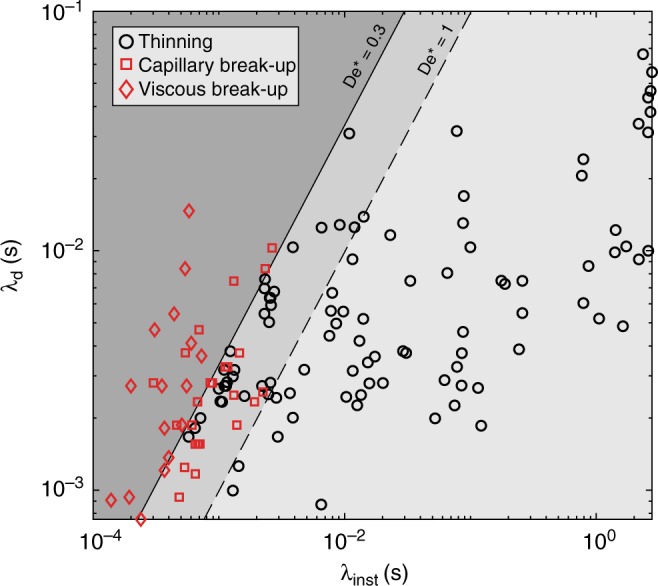
A behaviour map for the fluid dynamic break-up of low-viscosity liquids under extension. All experiments are plotted and colour coded for thinning (black) and break-up (red). The style of break-up is denoted by the symbol type, where the squares and diamonds correspond to capillary and viscous break-up, respectively. For modified Deborah numbers (De*) >1, only thinning is observed, at De* <1 break-up is possible and at De* <0.3, only break-up is observed

Our experimental data show both viscous and capillary break-up occurs over a large range of deformation timescales (~2 orders of magnitude; Fig. 3). However, independent of the mechanism, liquids are observed to break-up when De* <1, as the instability timescale is shorter than the deformation timescale. This sets the failure criterion for magma fluid dynamic break-up as:

5 $$\left. {\begin{array}{*{20}{c}} {{\mathrm{Oh}} > 1:\frac{{\dot \gamma \eta d}}{\sigma }} \\ {{\mathrm{Oh}} < 1:\dot \gamma \sqrt {\frac{{\rho d^3}}{\sigma }} } \end{array}} \right\} < 1.$$

At 1>De*>0.3, there is a transitional zone where both thinning and break-up can occur; however, in our experiments at De* <0.3, only break-up is observed. At De >1 the instability timescales are slower than the deformation timescale, so the extending liquid only thins (Fig. 3). Lastly, it is important to note that previous experimental studies of silicate melt elongation^41–43^, which analysed the viscoelastic behaviour, used a fragmentation criterion form of the Deborah number, which relates to the viscous and elastic response of the material. We do not investigate viscoelastic effects here as these are most relevant to higher viscosity melts deformed at higher strain rates^16^. For example, one fragmentation criterion that applies to higher viscosity (viscoelastic) melts is met when the strain rate exceeds 10^−2^*G*/*η*_s_, where *G* is the shear modulus at infinite frequency [Pa] and *η*_s_ is the shear viscosity [Pa·s] of the melt at zero frequency^7,8,41^.

**Fig. 4:**
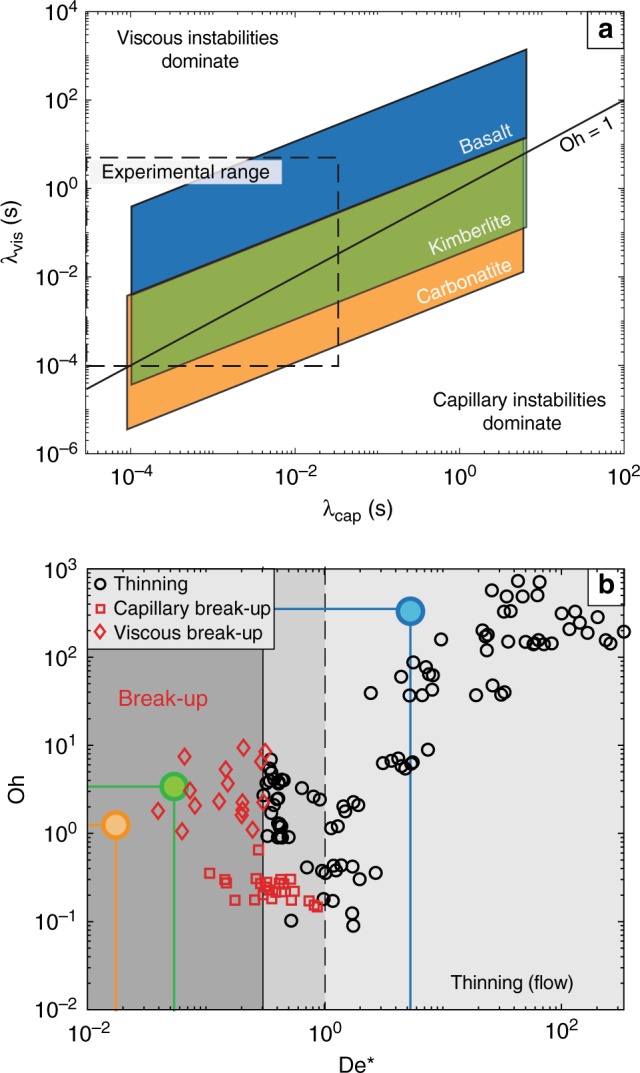
The break-up characteristics of natural volcanic melts under extension. **a** Predicted viscous and capillary timescales for volcanic pyroclasts. The shaded fields denote the range of Ohnesorge number expected for the eruption of basalt (blue), kimberlite (green) and carbonatite (orange). Note that the carbonatite field extends underneath the kimberlite field. A line of Oh = *λ*_vis_ / *λ*_cap _= 1 separates where capillary (Oh <1) or viscous (Oh >1) instabilities dominate. **b** Break-up vs. flow behaviour map that relates the intrinsic properties of the melt (Ohnesorge number; Oh) to the eruption conditions (modified Deborah number; De*). Shading from light, intermediate to dark grey indicates zones of solely thinning, both thinning and break-up and solely break-up, respectively. Lines show example calculation results for basalt (blue), kimberlite (green) and carbonatite (orange)

Now let us consider the break-up of volcanic low-viscosity liquids. Here we focus on basalts, kimberlites and carbonatites; however, our analysis is equally applicable to other high temperature melts (e.g. komatiites) or alkalic melts (e.g. nephelinites), for example. For reasonable estimates of physical melt properties (Table 1), these low-viscosity liquids can be subject to both viscous and capillary instabilities (Fig. 4a). The shorter the instability timescale, the more likely break-up will occur—enough time must be available during deformation and before clast cooling. Therefore, kimberlites and carbonatites are more prone to undergo fluid dynamic-induced break-up relative to a higher viscosity basalt. Furthermore, we predict that basaltic melts break up solely in the viscous regime, while kimberlitic and carbonatitic melts straddle the divide and can break-up by both capillary and viscous instabilities (Fig. 4a).

**Table 1:** Physical property data for the melts of basaltic, kimberlitic and carbonatitic composition used in this study

Parameter:	Basalt^54–56^			Kimberlite^36,57^			Carbonatite^58–60^		
	Min.	Max.	Best estimate	Min.	Max.	Best estimate	Min.	Max.	Best estimate
*d* [m]	10^−4^	10^−1^	–	10^−4^	10^−1^	–	10^−4^	10^−1^	–
*σ* [N m^−1^]	0.07	0.25	0.16	0.07	0.25	0.16	0.07	0.25	0.16
*ρ* [kg m^−3^]	2400	2800	2600	2500	2800	2650	2000	2300	2150
*η* [Pa·s]	10^1^	10^3^	10^2^	10^−1^	10^1^	10^0^	10^−2^	10^1^	10^−0.5^

The resulting parameter space shown in Fig. 4a was calculated by combining the values in Table 1 to calculate the limiting values of *λ*_cap_ and *λ*_vis_. Best estimate values will be used later to calculate the break-up of volcanic pyroclasts during fountaining

To demonstrate how our break-up criterion can be applied to volcanic systems, we relate the intrinsic properties of the system (defined by Oh; Eq. 3) to the extrinsic eruption variables (De*), where break-up can occur at De* <1 (Fig. 4b). This criterion can be applied to any low-viscosity liquid that forms a thinning filament by elongation. Hence, it is applicable to many processes (Fig. 1) that occur during Hawaiian- and Strombolian-type eruptions. Additionally, we emphasise that break-up can occur at De* <1, irrespective of the melt properties (Oh group; Fig. 4b). Now as an example of how our analysis can be used, let us consider a pyroclastic jet or fountaining episode containing spherical clasts 5 cm in diameter, *d*_0_. The typical velocity difference^44^ (Δ*v*) between the pyroclast and gas is ~100 m s^−1^, which acts to stretch clasts at a rate, $\dot \gamma$, calculated as^32,45,46^:

6 $$\dot \gamma = \frac{{\Delta v}}{{d_0}}\sqrt {\frac{{\rho _{\mathrm{g}}}}{\rho }},$$

where $\rho _{\mathrm{g}}$ is the density of the ambient gas within the fountain. As an example, we take the tread thickness at potential break-up *d* to be 200 μm^47^. Performing this analysis on pyroclasts of basaltic, kimberlite and carbonatitic composition proves highly instructive (Fig. 4b; best estimate values, Table 1). Under these conditions, we find that basaltic pyroclasts do not break but would extend to form thin (~200 μm) melt strands such as Pele’s hair—a common product of basaltic hawaiian lava fountaining episodes^31,47^. Kimberlitic and carbonatitic pyroclasts would undergo further break-up under the same eruption conditions creating smaller pyroclasts and potentially producing a melt spray (e.g. atomisation), which would coat crystals and lithic fragments during eruption^33,36^.

In volcanic systems pure melts (the focus of this study) are not always present. Rather, magma exists—a multiphase suspension composed of a melt (the liquid phase with Newtonian viscosity) that suspends bubbles (gas) and/or crystals (solid). The addition of both of these phases can introduce a non-Newtonian rheology^48^. Crystals increase the magma viscosity and can introduce a yield stress once the particle concentration is sufficient for particle–particle interactions to occur^48^. The onset of a yield stress has been reported to occur from particle concentrations of ~0.25–0.55 vol.%; however, this value depends on the particle size and aspect ratio^49^. A recent experimental study^15^ has shown that the presence of crystals promotes brittle fracture during filament extension. This was due to non-Newtonian effects at high deformation rates.

The presence of bubbles within the extending liquid is likely to introduce many complicated effects. For example, when bubbles do not deform during flow, they act to increase the viscosity of the suspension^50^. Hence, they could suppress viscous break-up by extending the viscous instability timescale (Eq. 2). Alternatively, the diameter controlling break-up might be reduced to the film thickness between bubbles, rather than the filament diameter. This would act to reduce both the viscous and capillary instability timescales, and introduce nucleation points for crack development^51^. In the presence of several bubbles the liquid is likely to experience enhanced necking and sudden rupture above a critical flow rate^52^. Foams have been shown to rupture in three ways under extension^51^. Viscous break-up to a central pinch point was observed at the lowest critical velocities, while brittle fracture occurs at larger velocities, where foams break apart from fracture points within the filament. A transition zone at intermediate rates is observed where both break-up mechanisms are apparent. This transition zone reduces in size with increasing width of the initial filament. It is therefore clear that when bubbles and/or crystals are suspended in the melt, non-Newtonian effects are likely to change the characteristics of extensional break-up. This should be the focus of future research.

Our experiments and scaling analysis have shown that low-viscosity volcanic melts are susceptible to fluid dynamic break-up—a fundamentally different phenomenon to classical brittle fragmentation^1^. Fluid dynamic-induced break-up is supported by melts with high surface tension and low shear viscosities that are stretched into thin filaments. These insights enable a volcanologist to interpret deposits (e.g. Pele’s hair vs. melt-coated crystals) from historic eruptions to back-calculate their formation conditions, or to predict the evolution of eruptive processes (Figs. 1, 4), based on the knowledge of the deformation conditions (De*) and the melt properties (Oh). Therefore, the fragmentation criterion presented here (Eq. 5) takes us closer to answering a fundamental question in volcanology—will an eruption behave in an effusive or an explosive manner?

## Methods

### CaBER measurements

The Capillary Breakup Extensional Rheometer (CaBER) was fitted with parallel plates with a diameter of 2, 4, 5 or 8 mm and a separation between 0.5 and 4 mm. The sample was loaded between the plates and a step strain was applied with a strike distance between 2.5 and 17.5 mm and a strike time between 20 and 250 ms. The conditions used for each experiment are shown in the Supplementary Data 1. The initial strike (i.e. the deformation) and subsequent relaxation was filmed using a camera at 550 frames per second. The experiments were performed at room temperature. The temperature of the fluid was monitored for each experiment and was always in the range 18.45–19.10 °C. The extracted video was monitored and the filament diameter (*d*) measured from the last frame in which the filament was uniform (i.e. in the case of break-up, the frame before necking and break-up occurred or in the case of thinning, the final frame of the strike).

The strain rate ($\dot \gamma$) experienced by the extending filament was calculated as:

7 $$\dot \gamma = - \frac{2}{d}\frac{{\Delta d}}{{\Delta t}},$$

where, as previous, *d* is the filament diameter measured from the last frame in which the filament was uniform. Here, the extension rate, $\dot \gamma$, was calculated immediately before break-up or the end of strike. Specifically, Δ*d* was calculated by comparing the filament diameter one frame before break-up or end of strike and therefore Δ*t* is the time step of one video frame (~1.8 ms).

### Materials

The analogue fluid used in this study was a Newtonian sugar solution manufactured by Tate and Lyle and termed “golden syrup” hereafter. To produce a wide range of working fluid viscosities, the golden syrup was diluted to 0, 0.5, 1, 2, 5, 10, 20, 25, 30 and 40 wt.% deionized water.

### Density measurements

Density values for the range of analogue fluids were measured using a calibrated 50 ml pycnometer flask. Fluids were added to the flask and left to equilibrate at a range of temperatures between 10 and 30 °C within a temperature-controlled water bath. Once equilibrated, the flask exterior was dried and then its mass measured on a ±500 μg desktop balance. This procedure was performed on aqueous dilutions of golden syrup (2, 5, 10, 15, 20, 25, 30 and 40wt.% H_2_O). The density of a particular fluid was found to vary as a function of temperature and follows the purely empirical general expression:

8 $$\rho = AT + B,$$

where *T* [°C^−1^] is the temperature and *A* [kg m^−3^ °C^−1^] and *B* [kg m^−3^] are fitted constants (Table 2). For each experiment the measured temperature, *T*, was used to calculate the fluid density.

**Table 2:** Density fitting constants for golden syrup dilutions used

Dilution (wt.%)	*A* [kg m^−3^ °C^−1^]	*B* [kg m^−3^]
0	−0.6094	1450.7
2	−0.5996	1439.4
5	−0.5965	1423.1
10	−0.5935	1396.5
15	−0.5942	1389.4
20	−0.5817	1343.8
25	−0.6727	1337.0
30	−0.4545	1309.9
40	−0.4615	1262.2

The density of a golden syrup dilution can be described as function of temperature by the expression: *ρ* = *AT*+*B* (Eq. 8), where *T* [°C^−1^] is the temperature and *A* [kg m^−3^ °C^−1^] and *B* [kg m^−3^] are constants.

### Viscosity measurements

Viscosity measurements were conducted on a TA Instruments Discovery HR2 rheometer equipped with a Peltier plate and a 60 mm diameter, 2° cone with a gap of 53 μm. Samples were equilibrated at the desired temperature (same temperature as during CaBER tests) for 5 min before testing in the shear rate range between 0.1 and ≥50 s^−1^. The viscosity was calculated as an average over the measured shear rate range, once measurements had stabilized. The lowest viscosity samples, of 25, 30 and 40 wt.% H_2_O, were measured using a TA instruments AR2000 rheometer equipped with a double concentric cylinder with a gap of 500 μm. The testing procedure was the same as that used on the Peltier plate.

### Surface tension measurements

Surface tension measurements were conducted using a Krüss K10ST tensiometer, using the Du Noüy ring method^53^. The ring was attached to the tensiometer and allowed to hang freely, and the instrument zeroed. The ring was then lowered into the golden syrup solution until completely submerged, and the surface tension digital readout adjusted to ~10 mN m^−1^ below the estimated surface tension. The sample vessel was lowered by the servomotors, withdrawing the ring from the sample, until a maximum in force was reached. This was recorded as the surface tension. The reported surface tension was taken as an average of at least five measurements. The ring was cleaned by submerging in distilled water before being spun in the flame of a gas burner for ~10 s until it glowed red. The vessel speed control was altered depending on the viscosity of the sample, with higher viscosity samples requiring a lower speed setting. The pure golden syrup (i.e. 0% dilution) was too viscous for this technique, so a value of 0.08 N m^−1^ was taken from the published literature^50^.

### Estimation of natural instability timescales

The timescales of the natural systems shown in Fig. 4 were calculated using the literature values given in Table 1 and Eqs. 1 and 2. To extract the range of capillary instability timescales, the maximum and minimum values were calculated using the range of physical properties in Table 1. The only independent variable in the viscous instability timescale is the viscosity. Hence, varying the viscosity (within the literature range) gives the range of viscous instability timescales expected for each capillary timescale. Table 3 details these calculations.

**Table 3:** The instability timescales for natural volcanic compositions

*d* [m]	*σ* [N m^−1^]	*ρ* [kg m^−3^]	*η* [Pa·s]	*λ*_cap_ [s]	*λ*_vis_ [s]
Basalt					
0.0001	0.25	2400	10	9.80 × 10^−5^	4.00 × 10^−3^
0.1	0.07	2800	10	6.32	1.43 × 10^1^
0.0001	0.25	2400	1000	9.80 × 10^−5^	4.00 × 10^−1^
0.1	0.07	2800	1000	6.32	1.43 × 10^3^
Kimberlite					
0.0001	0.25	2500	0.1	1.00 × 10^−4^	4.00 × 10^−5^
0.1	0.07	2800	0.1	6.32	1.43 × 10^−1^
0.0001	0.25	2500	10	1.00 × 10^−4^	4.00 × 10^−3^
0.1	0.07	2800	10	6.32	1.43 × 10^1^
Carbonatite					
0.0001	0.25	2000	0.01	8.94 × 10^−5^	4.00 × 10^−6^
0.1	0.07	2300	0.01	5.73	1.43 × 10^−2^
0.0001	0.25	2000	10	8.94 × 10^−5^	4.00 × 10^−3^
0.1	0.07	2300	10	5.73	1.43 × 10^1^

This table lists the parameter values used to calculate the viscous and capillary instability timescales for basaltic, kimberlitic and carbonatitic pyroclasts

## Supplementary information

Description of Additional Supplementary Files

Supplementary Data 1

Supplementary Movie 1

Supplementary Movie 2

Supplementary Movie 3

## Data Availability

All data generated or analysed during this study are included in Supplementary Data 1.
