# Supplementary material for: Fluid dynamic induced break-up during volcanic eruptions
Source: Nat Commun. 2019 Aug 23;10:3828. doi: 10.1038/s41467-019-11750-4 (PMC6707319; doi:10.1038/s41467-019-11750-4)
Supplement: Supplementary file 1 — Description of Additional Supplementary Files [file 41467_2019_11750_MOESM1_ESM.pdf]

Description of Additional Supplementary Files

File Name: Supplementary Data 1
Description: A list of experiments performed and their conditions. This dataset describes all the experimental conditions and the corresponding fluid physical properties, calculated thread diameters, timescales and dimensionless parameters for all experiments performed.

File Name: Supplementary Movie 1
Description: Video of filament thinning. Representative video of a sample undergoing thinning. Fluid is golden syrup diluted with 5 wt.% water, with initial sample diameter of 4 mm and height of 2 mm. The sample was extended 14.65 mm over 0.1 s. The video has been slowed by a factor of 10.

File Name: Supplementary Movie 2
Description: Video of capillary break-up. Representative video of a sample undergoing capillary breakup. Fluid is golden syrup diluted with 30 wt.% water, with initial sample diameter of 5mm and height of 2.5 mm. The sample was extended 6.48 mm over 0.1 s. The video has been slowed by a factor of 10.

File Name: Supplementary Movie 3
Description: Video of viscous break-up. Representative video of a sample undergoing viscous breakup. Fluid is golden syrup diluted with 20 wt. % water, with initial sample diameter of 4mm and height of 2 mm. The sample was extended 14.65 mm over 0.1 s. The video has been slowed by a factor of 10.
